# Abnormal subchondral bone remodeling and its association with articular cartilage degradation in knees of type 2 diabetes patients

**DOI:** 10.1038/boneres.2017.34

**Published:** 2017-11-07

**Authors:** Yan Chen, Yong-Can Huang, Chun Hoi Yan, Kwong Yuen Chiu, Qingjun Wei, Jingmin Zhao, X Edward Guo, Frankie Leung, William W Lu

**Affiliations:** 1 Department of Bone and Joint Surgery, The First Affiliated Hospital, Guangxi Medical University, China; 2 Department of Orthopaedics and Traumatology, The University of Hong Kong, Hong Kong; 3 Bone Bioengineering Laboratory, Department of Biomedical Engineering, Columbia University, New York, NY, USA; 4 Shenzhen Engineering Laboratory of Orthopaedic Regenerative Technologies, Orthopaedic Research Center, Peking University Shenzhen Hospital, Shenzhen, China

## Abstract

Type 2 diabetes (T2D) is associated with systemic abnormal bone remodeling and bone loss. Meanwhile, abnormal subchondral bone remodeling induces cartilage degradation, resulting in osteoarthritis (OA). Accordingly, we investigated alterations in subchondral bone remodeling, microstructure and strength in knees from T2D patients and their association with cartilage degradation. Tibial plateaus were collected from knee OA patients undergoing total knee arthroplasty and divided into non-diabetic (*n*=70) and diabetes (*n*=51) groups. Tibial plateaus were also collected from cadaver donors (*n*=20) and used as controls. Subchondral bone microstructure was assessed using micro-computed tomography. Bone strength was evaluated by micro-finite-element analysis. Cartilage degradation was estimated using histology. The expression of tartrate-resistant acidic phosphatase (TRAP), osterix, and osteocalcin were calculated using immunohistochemistry. Osteoarthritis Research Society International (OARSI) scores of lateral tibial plateau did not differ between non-diabetic and diabetes groups, while higher OARSI scores on medial side were detected in diabetes group. Lower bone volume fraction and trabecular number and higher structure model index were found on both sides in diabetes group. These microstructural alterations translated into lower elastic modulus in diabetes group. Moreover, diabetes group had a larger number of TRAP^+^ osteoclasts and lower number of Osterix^+^ osteoprogenitors and Osteocalcin^+^ osteoblasts. T2D knees are characterized by abnormal subchondral bone remodeling and microstructural and mechanical impairments, which were associated with exacerbated cartilage degradation. In regions with intact cartilage the underlying bone still had abnormal remodeling in diabetes group, suggesting that abnormal bone remodeling may contribute to the early pathogenesis of T2D-associated knee OA.

## Introduction

Type 2 diabetes (T2D) is an exceedingly common chronic metabolic disorder that affects over 387 million adults worldwide and is projected to reach 592 million by 2035.^1^ T2D affects a number of organs, including the skeleton. T2D patients have increased fragility fracture (for example, at femoral neck, distal radius, and tibia) induced by bone loss and deficits of bony microarchitecture and strength.^2–5^ It was evident that bone loss was attributable to increased bone resorption^6^ and decreased osteoblastogenesis.^7^ In addition, the disruption of bony microarchitecture partly accounts for strength deficits in T2D patients.^4^

It has been reported that T2D is associated with significantly increased prevalence of osteoarthritis (OA),^8^ the most common degenerative joint disease and the major cause of physical disability in adults. In longitudinal cohort studies, T2D has been determined as an independent risk factor of knee OA,^9^ and the concept diabetic OA has been proposed.^10^ However, the underlying mechanism by which T2D contributes to knee OA pathogenesis is largely unknown. As articular cartilage degradation has long been regarded as the primary characteristic of knee OA, recently the catabolic effect of high concentrations of glucose and insulin on cartilage through molecular and cellular mechanisms was the major explanation for the relationship between T2D and knee OA.^10–13^ But most of these data were obtained from cellular or animal models, and the relationship has not been validated in humans yet.

Moreover, currently OA has been seen as a “whole joint disease”, with all joint tissues, including the cartilage, subchondral bone, etc., being involved in the^14,15^ pathogenesis. It was reported that focally increased subchondral bone remodeling and impaired structure of lead to altered mechanical properties, thereby adversely affecting the overlying cartilage.^14,16,17^ Subchondral bone sclerosis, characterized by increased bone density, is associated with OA cartilage degradation.^18,19^ In turn, another subtype of OA subchondral bone, characterizing by abnormal remodeling, distinctively decreased density and deteriorated structure, has been recognized as a contributor to cartilage degeneration.^20,21^ However, the changes in subchondral bone remodeling, microstructure and strength in knees from T2D patients and their relationship with cartilage degeneration have not yet been fully understood.

Thus, to unravel the relationship of T2D with subchondral bone remodeling and knee OA progression, the aims of this study were: (1) to determine whether subchondral bone remodeling, microstructure and strength and cartilage morphology are altered in knees from patients with T2D; and (2) to examine the associations of the bony alterations with cartilage degradation. It was hypothesized that increased subchondral bone remodeling led to deteriorated microstructure and strength, which in turn aggravated cartilage degradation in T2D patients.

## Materials and methods

### Subjects

This study was approved by the Institution Review Board of the University of Hong Kong (Ref No.: UW-09368). Each patient provided written informed consent prior to their participation in the study.

A series of 121 patients who had been diagnosed with primary knee OA and underwent total knee arthroplasty (TKA) were recruited from April 2014 to February 2016 (The University of Hong Kong). The patients were divided into non-diabetic (*n*=70) and diabetes (*n*=51) groups according to the status of T2D. The diagnosis of OA and T2D was based on the American College of Rheumatology criteria^22^ and the American Diabetes Association criteria (that is, glycated hemoglobin A1c >6.5% or fasting plasma glucose >7.0 mmol·L^−1^ or 2 h plasma glucose >11.1 mmol·L^−1^ during an oral glucose tolerance test),^23^ respectively. The details of inclusion criteria were described previously.^17,24^ Briefly, all subjects are Southern Chinese in Hong Kong aging between 46 and 78 years, and all female subjects were amenorrheic for at least 6 months. Exclusion criteria were subjects with a history of knee joint trauma, other arthritis, osteoporosis, metabolic bone disease, bone tumor, primary or secondary hyperparathyroidism, smoking or alcoholism or type 1 diabetes, or patients taken any medications affecting bone remodeling (for example, bisphosphonates, estrogen, selective estrogen receptor modulator, or diabetic medication thiazolidinediones).

### Clinical data

Patients’ demographic data such as age, gender, and body mass index (BMI) were collected. The radiographs of OA knee were evaluated using Kellgren and Lawrence (K-L) system by an experienced reader (FL) blind to the diabetes status.^25^ Mechanical alignment of the lower extremity (using the hip-knee-ankle angle) was determined by drawing lines connecting the hip, knee, and ankle joint centers, which were defined as the center of the femoral head, center of the femoral condyles, and midpoint of the medial and lateral margins of the ankle, respectively.^26^ The knee function was assessed using the American Knee Society Score system, which includes the Knee Society Knee Score (pain, stability, range of motion, and so on) and Knee Society Functional Score (walking, stairs, and aids).^27^ Obesity was defined as BMI≥30 kg·m^−^^2^ according to the World Health Organization criteria.^28^ Other comorbidities such as hypertension and vascular diseases were determined by clinical records. The average of hemoglobin A1c over the previous 8 years was calculated from clinical records and used as an indicator of past glycemic control. The use of insulin and oral T2D medications was ascertained. Furthermore, blood samples were drawn after an overnight fast before TKA. The following biochemical parameters were measured by standard methods: hemoglobin A1c, fasting plasma glucose, erythrocyte sedimentation rate, cholesterol, triglycerides, high-density lipoprotein cholesterol, and low-density lipoprotein cholesterol.

### Micro-computed tomography (Micro-CT)

**Figure 1:**
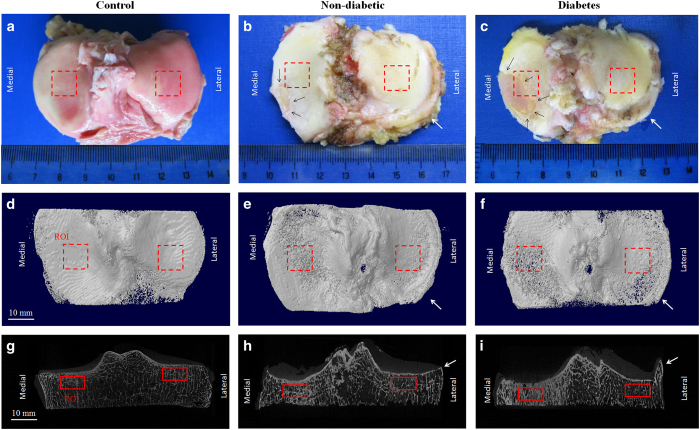
Macroscopic and micro-CT images of tibial plateaus from non-diabetic and diabetes patients. Macroscopic images were shown in (**a**–**c**). Black arrows (**b**,**c**) indicated edges of the remained cartilage in OA specimens. The corresponding micro-CT images were displayed in (**d**–**f**) (top view) and (**g**–**i**) (coronal view), with the red rectangles indicating the ROIs of subchondral bone (solid lines) and subchondral plate (dashed lines) on medial and lateral sides. White arrows (**b**,**c**,**e,f,h**,**i**) indicate osteophytes in non-diabetic and diabetes groups.

Tibial plateau specimens (*n*=121) were collected from the 121 patients during TKA (Figure 1b and c). Furthermore, tibial plateaus from cadaveric donors (*n*=20, 13 females and seven males, age 66±5 years old; one tibial plateau was attained from each cadaveric donor from the University of Hong Kong) without T2D, OA, or bone diseases were collected as controls (Figure 1a). The specimens were scanned using a micro-CT scanner (Bruker MicroCT 1076, Luxemburg, Belgium) with established protocols.^24^ Briefly, the following scanning parameters were used: 17.3 μm isotropic voxel size, 55 kV voltage, 109 μA current, 200 ms integration time and 4 000 projections. The two-dimensional images were converted into discrete binary objects by the global thresholding and binarisation procedures using the software CTAn (Bruker microCT). For three-dimensional analysis, the volume of interest was selected as 10×10×5 mm^3^ of trabecular bone beneath the subchondral plate at the center of condyles (Figure 1d–i), as described.^24^ Briefly, the subchondral bone and subchondral plate were separated with the aid of the edge detection function of MAT-LAB R2010a (MathWorks, Massachusetts, USA). After segmentation, the irregular boundary of binary objects was detected. The edges of subchondral bone were saved as the region of interest in the binary bitmap images, and unwanted edges were removed based on their coordinates in the segmented images to obtain the region of interest for analysis. In addition, a second region of interest, the subchondral plate overlying the selected subchondral bone, was also chosen for analysis, as described.^24^

For subchondral bone, the following parameters were calculated using CTAn: bone volume fraction (BV/TV), trabecular number (Tb.N), structure model index (SMI), trabecular separation (Tb.Sp), trabecular thickness (Tb.Th) and connectivity density (Conn.D). Moreover, bone mineral density (BMD) of subchondral bone was calibrated by using the attenuation coefficient of two hydroxyapatite phantoms (supplied by Bruker microCT) with defined BMD of 0.25 and 0.75 g·cm^−3^.^17^ In addition, for evaluation of subchondral plate structure, subchondral plate thickness (Pl.Th) and porosity (Pl.Po) was analyzed as described.^24^ Briefly, Pl.Th was given by: Pl.Th=tissue volume/subchondral plate area (that is, 10×10 mm^2^). The micro-CT three-dimensional images and the BMD color maps were created using the softwares CTvol and CTvox (Bruker microCT), respectively.

### Micro-finite element analysis (FEA)

Each thresholded micro-CT image of the subchondral bone was converted to a micro-FE model by converting each voxel to an eight-node brick element. Bone tissue was modeled as an isotropic, linear elastic material with a Young’s modulus of 15 GPa and a Poisson’s ratio of 0.3.^29^ A uniaxial compression test along the longitudinal axis of tibia was simulated up to 1% apparent strain. A custom FEA solver (FAIM, version 7.1; Numerics88, Calgary, AB, Canada) on a desktop workstation (Linux CentOS 7.1, 2×6-core Intel Xenon, 64 GB RAM, Columbia University) was used to solve the models as previously described.^30^ Elastic modulus, which characterizes the mechanical properties of subchondral bone and is closely related to bone strength,^31^ was calculated from the linear FEA simulation.

### Histology

After the micro-CT scan, the tissue plugs corresponding to the volume of interests of tibial plateaus (*n*=20 in non-diabetic and diabetes groups, respectively; specimens were randomly selected; *n*=20 in control group) were processed for histological evaluation. Serial sections (5 μm) were stained with Safranin O and Fast Green. Cartilage degradation was evaluated using the Osteoarthritis Research Society International (OARSI) scoring system^32^ by experienced cartilage pathologists (WZ), as previously described. The observer was blinded with respect to the category (control, non-diabetic, or diabetes group) and macroscopic description of the specimens. A partial score for each scale category (structure abnormalities, cellularity, matrix staining, and tidemark integrity) was allocated, and they were combined to get the average scores for every section.

### Immunohistochemistry

Tissue sections (*n*=20 in non-diabetic and diabetes groups, respectively; specimens were randomly selected; *n*=20 in control group) from lateral tibial plateau were evaluated using immunohistochemistry as described previously.^14,17^ In brief, biomarker of the osteoclasts (tartrate-resistant acidic phosphatase, TRAP) was detected using TRAP staining with a commercial TRAP kit (Sigma-Aldrich, Missouri, USA). To detect biomarkers of osteoblasts (osteocalcin) and osteoprogenitors (osterix),^14,17^ sections underwent heat-induced antigen retrieval in citrate buffer, followed by incubation with either anti-osterix (Abcam, Cambridge, UK) or anti-osteocalcin (TakaRa, Shiga, Japan) primary antibodies overnight. Next, horseradish peroxidase-labeled secondary antibodies (Abcam) was added and incubated for 60 min. Color was developed using diaminobenzidine (DAB) as substrate (Vector Lab, California, USA). After images were captured, the number of positive stained cells was quantified as previously described.^14,17^ Briefly, five sequential sections from each sample were stained and for each section, five areas were measured.^14,17^

### Statistical analysis

The clinical, micro-CT, micro-FEA, histology and immunohistochemistry data were tested for normality using Shapiro–Wilk Test. The comparison of clinical data between non-diabetic and diabetes groups was conducted using Student’s *t*-test for normally distributed variables, Mann–Whitney *U-*test for nonparametric variables and *χ*^2^-test for categorical data. The comparison of micro-CT, micro-FEA, histology and immunohistochemistry variables among the control, non-diabetic and diabetes groups was performed using one-way analysis of variance (ANOVA). If the result was statistically significant, a *post-hoc* test was further carried out. Statistical significance was set as an alpha level less than 0.05. SPSS 20.0 (Chicago, IL, USA) and used for all statistical analyses.

## Results

### Clinical data

No statistically significant difference was found regarding the patients’ age, gender, BMI, K-L grade, mechanical alignment, tryglycerides, an so on between the non-diabetic and diabetes groups (*P*>0.05, Table 1). Diabetes group had obviously higher hemoglobin A1c (11.1%, *P*=0.032) and fasting plasma glucose (52.4%, *P*=0.003) than non-diabetic group. The complete information of current and past antidiabetic medications was listed in Supplementary table 1.

### Histology

**Figure 2:**
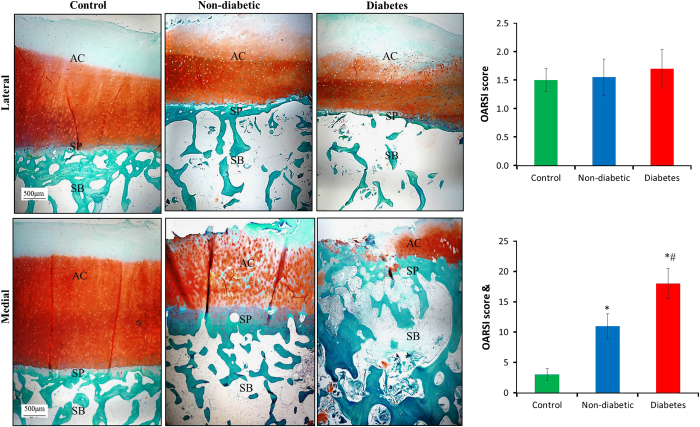
Histological changes of cartilage and subchondral bone from non-diabetic and diabetes patients. Cartilage damage was not obviously observed in all the groups on lateral tibial plateau. The analysis showed that there were no statistically significant differences in OARSI score among groups. On medial side, diabetes group showed more obvious disruption of cartilage surface and loss of proteoglycans, and these degenerative changes extended into deeper zone than non-diabetic group. The analysis showed that there were statistically significant differences in OARSI score among groups. *Post-hoc* tests revealed a significantly higher cartilage OARSI score in diabetes group when compared with non-diabetic group. ^&^*P*<0.05 among control, non-diabetic and diabetes groups according to one-way ANOVA. ^#^*P*<0.05 vs non-diabetic group and *P<0.05 vs control group according to *Post-hoc* tests. AC, articular cartilage; SB, subchondral bone; SP, subchondral plate.

Results of Safranin O and Fast Green staining were shown in Figure 2. On lateral side, cartilage damage was not obviously observed in all the groups. The analysis showed that there were no statistically significant differences in OARSI score among groups (1.5±0.2 for control group, 1.6±0.3 for non-diabetic group, and 1.7±0.3 for diabetes group, *P*=0.72, Figure 2). On medial side, diabetes group displayed more obvious disruption of cartilage surface and loss of proteoglycans, and these degenerative changes extended into deeper zone than non-diabetic group (Figure 2). The analysis showed that there were statistically significant differences in OARSI score among groups (3±1 for control group, 11±2 for non-diabetic group and 18±2.3 for diabetes group, *P*=0.002). *Post-hoc *tests revealed a significantly higher cartilage OARSI score in diabetes group when compared with non-diabetic group (63.6%, *P*=0.008).

### Micro-CT and micro-FEA

**Figure 3:**
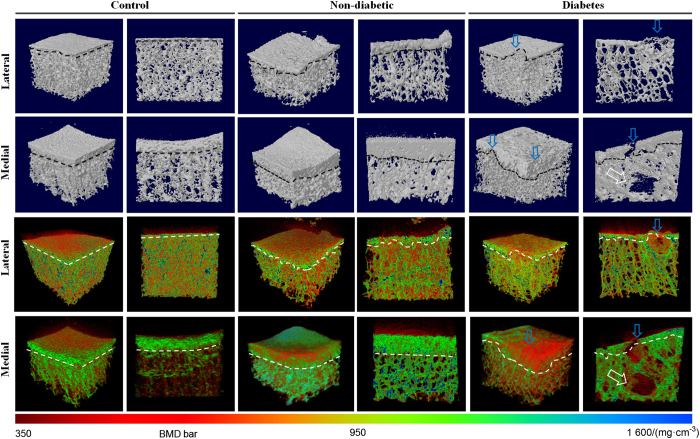
Micro-CT three-dimensional images and BMD maps of subchondral bone from non-diabetic and diabetes patients. The dashed black and white lines indicate the boundaries of subchondral bone. Note the bone lesions (white arrows) at subchondral bone and disruption (blue arrows) of subchondral plate in diabetes group. The BMD bar is displayed in the bottom.

Micro-CT three-dimensional images and BMD color maps of subchondral bone and subchondral plate were showed in Figure 3. On lateral side of the tibial plateaus, the one-way ANOVA shows that there were statistically significant differences in BV/TV, Tb.N, SMI, Tb.Sp, and Conn.D of subchondral bone among groups (*P*<0.003, Table 2). Post-hoc tests revealed that diabetic group had lower BV/TV (−31.4%, *P=*0.003), Tb.N (−13.9%, *P=*0.013), and Conn.D (−32.1%, *P=*0.002), while significantly higher Tb.Sp (9.7%, *P=*0.047) and SMI (13.0%, *P=*0.011) at subchondral bone than those from non-non-diabetic group. No statistically significant differences in Tb.Th, DA, BMD, Pl.Th, Pl.Po or elastic modulus (*P*>0.144) of the subchondral bone were found among groups.

On medial side of tibial plateaus, the one-way ANOVA shows that there were statistically significant differences in BV/TV, Tb.N, SMI, Tb.Sp, Tb.Th, and BMD of subchondral bone (*P*<0.033, Table 3), and in Pl.Th and Pl.Po of subchondral plate among groups (*P*<0.036). Post-hoc tests revealed that diabetic group had lower BV/TV (−20.5%, *P=*0.005), Tb.N (−14.2%, *P=*0.013), Tb.Th (−16.8%, *P=*0.025), and BMD (−17.2%, *P=*0.003), while significantly higher Tb.Sp (13.5%, *P*<0.001) and SMI (111.8%, *P*<0.001) at subchondral bone than those from non-diabetic group. In addition, diabetes group had obviously lower Pl.Th (−27.1%, *P*<0.001) and higher Pl.Po (26.0%, *P*<0.001) at subchondral plate than non-diabetic group (Table 3). The micro-FEA results showed that there were obvious differences in elastic modulus among groups (*P=*0.033). *Post-hoc* tests revealed that the subchondral bone of diabetes group had much lower elastic modulus than non-diabetic group (−36.7%, *P*<0.001).

### Immunohistochemistry

**Figure 4:**
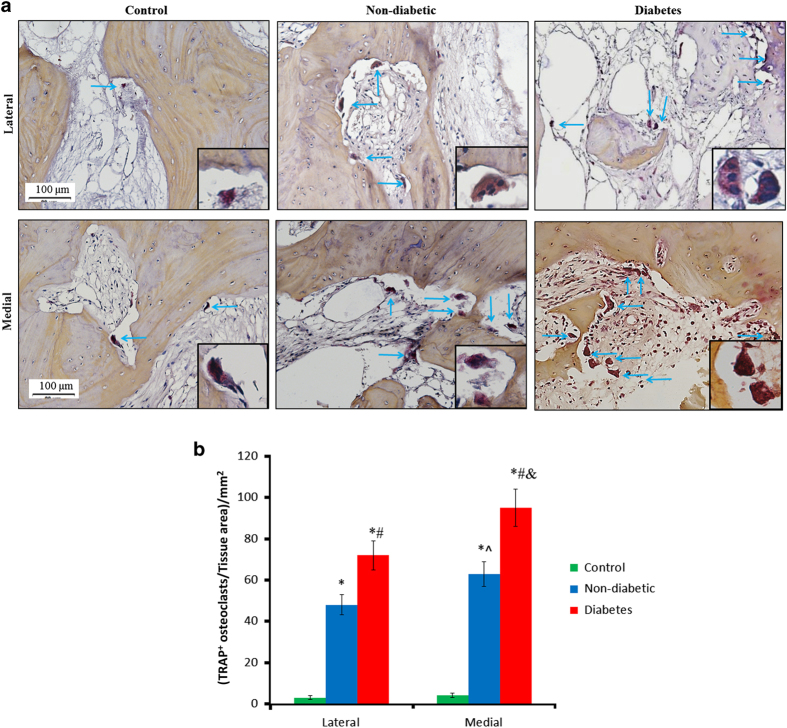
Activity of TRAP^+^ osteoclasts in subchondral bone from non-diabetic and diabetes patients. (**a**) Diabetes group generated larger bone marrow cavities than non-diabetic group on both lateral and medial sides. (**b**) One-way ANOVA analysis showed that there were significant differences in TRAP^+^ osteoclasts among groups on both sides. Of note, the number of TRAP^+^ osteoclasts in diabetes group was higher than non-diabetic group. In addition, the numbers of TRAP^+^ osteoclasts on medial sides were higher than lateral sides in both non-diabetic and diabetes group. Insert: morphology of TRAP^+^ osteoclasts. ^#^*P*<0.05 vs non-diabetic group and *P<0.05 vs control group on the same side according to *Post-hoc* tests; ^^^*P*<0.05 between lateral and medial sides in non-diabetic group; ^&^*P*<0.05 between lateral and medial sides in diabetic group.

**Figure 5:**
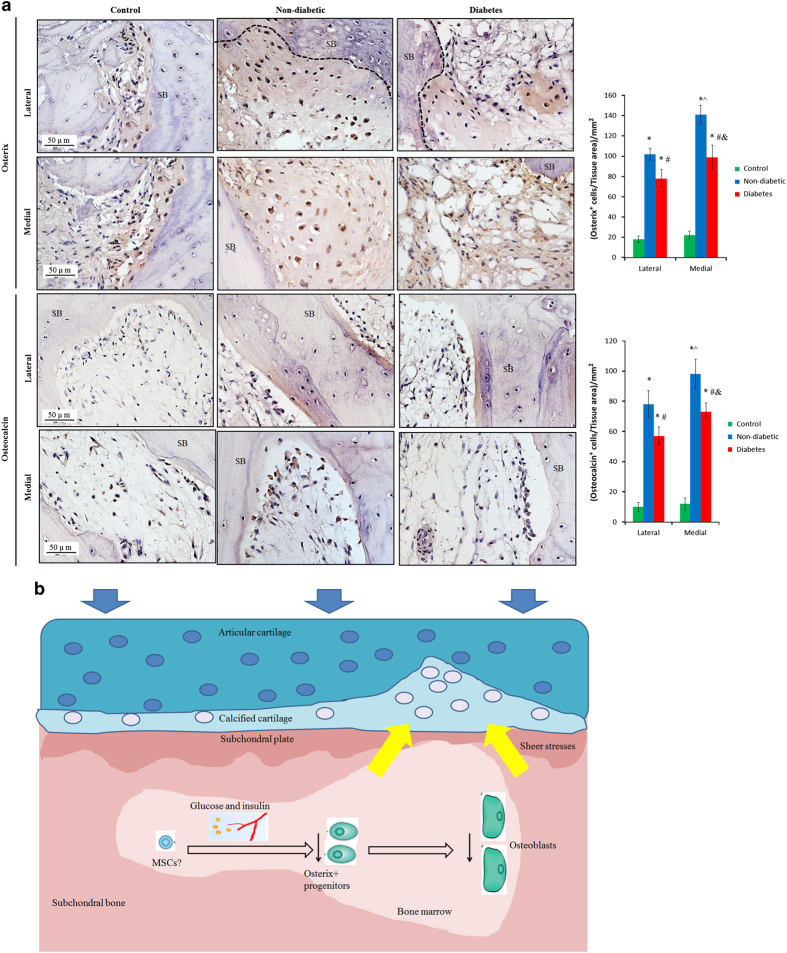
Activity of Osterix^+^ osteoprogenitors and Osteocalcin^+^ osteoblasts in subchondral bone from non-diabetic and diabetes patients**.** (**a**) The expression of Osterix^+^ osteoprogenitors and Osteocalcin^+^ osteoblasts was weaker in diabetes group than non-diabetic group on both lateral and medial sides. One-way ANOVA analysis showed that there were significant differences in the numbers of Osterix^+^ osteoprogenitors and Osteocalcin^+^ osteoblasts among groups. Of note, diabetes group had lower number of Osterix^+^ osteoprogenitors and Osteocalcin^+^ osteoblasts than non-diabetic group. ^#^*P*<0.05 vs non-diabetic group and *P<0.05 vs control group on the same side according to *Post-hoc* tests. ^^^*P*<0.05 between lateral and medial sides in non-diabetic group; ^&^*P*<0.05 between lateral and medial sides in diabetic group. SB, subchondral bone. (**b**) Schematic figure of the potential mechanism of abnormal subchondral bone remodeling in pathogenesis of T2D-induced knee OA: the hyperglycemia and hyperinsulinemia and/or the subsequent response in T2D have adverse effects on osteoprogenitors/mesenchymal stromal cells in subchondral bone, leading to impaired osteogenesis; meanwhile, the osteoclasts are activated, further contributing to abnormal bone remodeling. These changes lead to impairments of subchondral bone microstructure and strength, adversely affecting the overlying cartilage, resulting in knee OA.

Immunohistochemistry results were displayed in Figures 4 and 5. The analysis showed that there were statistically significant differences in the number of TRAP^+^ osteoclasts (*P*<0.026, Figure 4), Osterix^+^ osteoprogenitors (*P*<0.033, Figure 5) and Osteocalcin^+^ osteoblasts (*P*<0.039) among groups on both lateral and medial sides. *Post-hoc* tests revealed that there were a much larger number of TRAP^+^ osteoclasts in diabetes group than non-diabetic (50.5%, *P*<0.019, Figure 4) or control group. Meanwhile significantly lower numbers of Osterix^+^ osteoprogenitors (−30.75%, *P*=0.024) and Osteocalcin^+^ osteoblasts (−36.81%, *P*=0.031) were defined in the diabetes group than in non-diabetic group (Figure 5). In addition, the numbers of TRAP^+^ osteoclasts, Osterix^+^ osteoprogenitors and Osteocalcin^+^ osteoblasts on medial sides were higher than lateral sides in both non-diabetic and diabetes group (the increase>31.2%, *P*<0.02). There were no significant differences in the numbers of TRAP^+^ osteoclasts, Osterix^+^ osteoprogenitors or Osteocalcin^+^ osteoblasts between the two sides in control group (*P*>0.05).

## Discussion

In this study, we investigated changes in subchondral bone remodeling, microstructure and strength and their association with cartilage degradation in knees of T2D patients. We found that T2D patients had abnormal bone remodeling and microstructural deterioration and decreased strength. These bony changes were related with aggravated cartilage degradation. Furthermore, the bony changes have occurred in regions with intact cartilage. These findings suggest that abnormal subchondral bone remodeling may account for the exacerbation of cartilage damage when T2D and knee OA co-exist simultaneously in the same individuals.

Recently, the association between T2D and increased severity of OA has been partly explained by the degradative effect of high hyperglycemia and hyperinsulinemia on cartilage in cellular and animal studies.^10–13^ However, the effect has not been validated in human cartilage yet. In this study, we found a higher histological OARSI score on medial tibial plateau in diabetes group compared to non-diabetic group, which is consistent with previous findings.^11–13^ Interestingly, on lateral tibial plateau, statistically significant difference in histological OARSI was not found in non-diabetic or diabetes group compared to control group, or between non-diabetic or diabetes groups (Figure 2). Studies have demonstrated that some regions of the cartilage were still intact in lateral tibial plateaus from patients with advanced medial femorotibial OA who underwent TKA.^14,32,33^ These regions may be considered to represent early stage OA.^14,32,33^ Therefore, to better reveal the role of subchondral bone in diabetic OA pathogenesis, we also examined subchondral bone changes and their association with cartilage degradation on lateral side. Our results of lateral tibial plateau are consistent with previous study^14,33^ and suggest that the subchondral bone alterations induced by high hyperglycemia and hyperinsulinemia may be prior to cartilage degeneration in diabetic OA. However, this hypothesis should be tested in future studies.

The mechanism for the difference in OA severity between medial and lateral condyles is still not fully understood, although some studies have suggested that it may be related to the strength distribution pattern of the tibial condyles.^32,33^ In the current study, the differences detected in cartilage degeneration between medial and lateral sides of diabetic group further indicated an adverse effect of T2D on OA development.

Studies have demonstrated that the changes in subchondral bone also have an important role in OA pathogenesis.^14,18–21^ It is widely accepted that subchondral bone sclerosis is closely associated with cartilage degradation in OA.^17,19,34^ But besides this hypertrophic OA, another OA phenotype, the osteoporotic OA, characterized by distinctive decrease in subchondral bone density and increase in remodeling, has been proposed.^21,35^ It was reported that distinctively increased levels of subchondral bone remodeling and changed morphology were associated with more severe cartilage degeneration in patients with osteoporosis,^36^ and in animal models of ovariectomy-induced osteoporosis.^20,37^ Moreover, treatment with bone­acting agents improved subchondral bone structure and thus attenuated cartilage degeneration in both animal and human studies.^37–39^ These findings suggest that systemic factors (for example, osteoporosis) inducing subchondral bone impairments may have a role in OA pathogenesis. Nevertheless, these relationships are still not fully understood.

On the other hand, increased bone remodeling, microstructural impairment and fracture have been detected in a number of sites (for example, at femoral neck, distal radius, and tibia) in T2D patients.^1–5^ However, the changes in subchondral bone of T2D patients and their relationship with cartilage degradation have not been determined. We found in the present study that while no statistically significant difference in OARSI score was detected between non-diabetic and diabetes groups, increased bone remodeling and microstructural deteriorations were detected in diabetes group (Table 2). This indicates that in accordance with other bone sites,^1–5^ bony changes also occurred at subchondral bone; and that these changes may be prior to cartilage degradation and thus serves as an initiating factor for T2D-associated knee OA. Nevertheless, this proposition should be further investigated in future study.

Consistent with the bony changes on lateral side, subchondral bone on medial side displayed abnormal bone remodeling and microstructural deteriorations and decreased elastic modulus in diabetes group (Table 3), indicting an increased systemic bone remodeling in T2D. These changes were associated with more severe cartilage destruction, supporting the paradigm of focal interactions between subchondral bone and cartilage in pathogenesis of knee OA.^40^

The much higher SMI indicated that the trabeculae in diabetes group were markedly more rod-like compared with non-diabetic group. Rod-like trabeculae possesses lower mechanical strength and stiffness than plate-like trabeculae.^41^ To reveal bone strength more directly, micro-FEA technique, which accounts for the mechanical aspects of bone microstructure,^4^ was employed in this work. The results of FEA indicated that diabetes group had impaired subchondral bone strength, which may lead to decreased capacity to support the overlying cartilage and eventually result in subchondral bone collapse. Thus, the results from micro-CT, mechanical and histological analyses were consistent.

All the patients in our study were with advanced knee OA as defined by K-L grade (all in grade 3 or 4). However, even knees in K-L grade 4 do not necessarily indicate complete cartilage loss or true “end-stage” OA. It was reported that in K-L grade 4 knees, magnetic resonance imaging-detected cartilage loss and fluctuation of bone marrow lesions occurred frequently over a 30-month period.^42^ This suggests that K-L grade 4 knees can still progress and have different severity of cartilage and bony impairments in different pathogenic conditions. Thus, in our study, we could detect the difference in cartilage and bony changes in diabetes group compared to non-diabetic group.

Obesity is a strong risk factor for knee OA and it frequently coexists with T2D,^43^ and presence of obesity may be a major confounding factor for the association of T2D with cartilage and subchondral bone impairments. The prevalence of obesity varies among different ethnic groups. In the United States, ~72% of the T2D patients were obese,^43,44^ while in China obese patients comprise only ~7% of the whole T2D population.^45^ T2D patients in Japanese and South Korea showed a similar distribution of obesity with China.^44^ A weaker association between increasing BMI and diabetes in Asians compared with Caucasians was also noted.^46^ In the present study, the prevalence of obesity, BMI and laboratory parameters of lipid metabolism (that is, cholesterol, tryglycerides, high-density lipoprotein cholesterol, and low-density lipoprotein cholesterol)^28^ were compared between groups. No statistically significance in these parameters was found which may partly due to the relative low preference of obesity in the population of our study (Table 1).

The cellular mechanism underlying the association between T2D and bone remodeling has been investigated in a number of studies. In a rat model of non-obese T2D, reduction of bone formation rate was reported.^47^ Moreover, hyperglycemia promoted the increase of osteoclast formation and bone resorption as well as inflammation, mediated by reactive oxygen species and advanced glycation end-products.^48–50^ Our results of immunohistochemistry suggested that beyond the decrease of bone formation, increase of bone resorption also contribute to the lower bone volume in T2D. In addition, the differences of the results between the medial and lateral sides indicated an increase in subchondral bone remodeling in advanced diabetic OA compared to early diabetic OA.

Hence, our results may generate a model of “abnormal subchondral bone remodeling aggravating cartilage degradation” for the pathogenesis of T2D-induced knee OA (Figure 5c): the hyperglycemia and hyperinsulinemia and/or the subsequent response (increased levels of reactive oxygen species, glycation end-products, inflammatory mediators and so on) in T2D have adverse effects on osteoprogenitors/mesenchymal stromal cells in subchondral bone, leading to impaired osteoblastogenesis. Meanwhile, the osteoclasts are activated, further contributing to abnormal bone remodeling. These changes lead to impairments of subchondral bone microstructure and mechanical strength, adversely affecting the overlying cartilage, resulting in knee OA.

We should acknowledge that there are several limitations in this study. First, this is a cross-sectional study. Thus, the causality between impaired bony and cartilaginous structure in knee joints and T2D remains unclear, which could only be ascertained in future longitudinal study. Second, our specimens of tibial plateau were collected from knee OA patients, and we could not investigate specimens from individuals with T2D only. Nevertheless, it is generally difficult to attain tibial plateaus from patients with T2D only. Third, our study population was primarily consisted of patients with moderate-to-severe knee OA defined by K-L grade, the results of this study hence could not represent the conditions of early OA. In addition, the subchondral bone and cartilage samples were extracted from the center of the load-bearing area of the tibial plateaus. However, bone and cartilage from other regions of the tibial plateaus may differ. Last but not least, a number of diabetic medications have also been implicated in bone loss in T2D (for example, thiazolidinediones) and we have excluded diabetes patients on thiazolidinediones, but we could not exclude the T2D patients with additional use of diabetic medication. And diabetes patients may receive more medical attention than non-diabetic subjects, leading to increased awareness or even overtreatment (for example, use of painkillers) of OA. These may lead to an underestimation of OA severity (such as pain scores) in diabetes group.

In conclusion, our results determined that T2D patients have abnormal subchondral bone remodeling and microstructural impairments which were associated with the exacerbated cartilage degradation in knees. Hence, this study suggested that abnormal subchondral bone remodeling may be an underlying mechanism by which T2D aggravates knee OA.

## Figures and Tables

**Table 1:** Demographic and clinical characteristics of the patients without or with T2D

Parameters		Non-diabetic (*n=*70)	Diabetes (*n*=51)	*P*
Demographic	Gender (female *n*, %)^a^	48, 68.6	38, 74.5	0.671
	Age/year	73±8	70±7	0.530
	Body weight/kg	63.1±9.4	63.7±10.1	0.558
	Body height/cm	151.5±6.2	150.6±7.1	0.154
	BMI/(kg·m^−^^2^)	27.6±4.3	28.3±4.5	0.237
Sanding X-ray	K-L grade^a^			
	Grade 3	42	31	0.931
	Grade 4	28	20	
	Alignment of lower limb (degree)	169.6±12.5	163.6±8.8	0.116
Knee function	Knee Society Knee Score^b^	50 (43,57)	42 (36,48)	0.147
	Knee Society Functional Score^b^	49 (42,52)	44 (40,46)	0.225
Coexisting conditions	Obesity (BMI≥30 kg·m^−^^2^) (*n*, %)^a^	14 (20.0)	11 (21.6)	0.851
	Hypertension (*n*, %)^a^	38 (54.3)	39 (76.5)	0.188
	Vascular diseases (*n*, %)^a^	12 (17.1)	34 (66.7)	0.091
Biochemical tests	HbA1c/%	6.3±0.8	7.0±0.9	**0.032**
	FPG/(mmol·L^−1^)	6.3±1.9	9.6±4.1	**0.003**
Lipid metabolism	Cholesterol (<5.2 mmol·L^−1^)	4.6±1.0	4.9±1.0	0.372
	Tryglycerides (<1.7 mmol·L^−1^)	1.3±0.4	1.5±0.5	0.917
	HDL-C (≥1.04 mmol·L^−1^)	1.5±0.4	1.3±0.3	0.464
	LDL-C (≤3.10 mmol·L^−1^)	2.7±0.9	2.8±1.1	0.876

Abbreviations: BMI, bone mass index; FPG, fasting plasma glucose; HbA1c, glycated hemoglobin A1c; HDL-C, high-density lipoprotein cholesterol; K-L, Kellgren and Lawrence; LDL-C, low-density lipoprotein cholesterol; T2D, type 2 diabetes.

The comparisons of parameters were performed using student’s *t*-test and expressed as mean±s.d., except for the categorical data. Bold text indicates a statistically significant difference with a *P-*value<0.05.

a Using *χ*^2^-test and expressed as percentage (%), and the nonparametric variables.

b Using Mann–Whitney *U*-test and expressed as average (95% confidence interval).

**Table 2:** Comparisons of microstructure, BMD and strength of subchondral bone on lateral side in patients without or with T2D

Bony structure	Control (*n*=20)	Non-diabetic (*n*=70)	Diabetes (*n*=51)	*P*
(BV/TV)/%	21.74±4.08	18.35±4.15*	↓12.59±5.34*	**0.004**
Tb.N/mm^−1^	2.08±0.41	1.44±0.21*	↓1.24±0.28*	**0.003**
SMI	1.40±0.41	1.61±0.33*	↑1.82±0.3*	**0.034**
Tb.Sp/μm	392.68±65.75	485.64±71.33*	↑532.73±71.84*	**0.037**
Tb.Th/μm	119.46±14.63	149.81±21.03*	141.18±19.40*	0.605
Conn.D/mm^−^^3^	162.25±51.91	111.58±79.11*	↓75.80±58.73*	**0.034**
BMD/mg·cm^−3^	708.18±45.56	697.80±42.49	696.84±41.41	0.416
Pl.Th/mm	0.39±0.09	0.68±0.20*	0.66±0.19*	0.728
Pl.Po/%	21.37±9.5	26.46±7.96*	30.01±7.17*	0.144
Elastic modulus/MPa	296±107	225±124*	194±93*	0.682

Abbreviations: ANOVA, analysis of variance; BMD, bone mineral density; BV/TV, bone volume fraction; PI.Po, subchondral plate porosity; PI.Th, subchondral plate thickness; SMI, structure model index; T2D, type 2 diabetes; Tb.N, trabecular number; Tb.Th, trabecular thickness; Tb.Sp, trabecular separation.

The comparisons of microstructure parameters among the three groups were performed using one-way ANOVA and expressed as mean±s.d. A *post-hoc* test was further performed if the result was significant. Bold text indicates a statistically significant difference with a *P-*value<0.05. *P<0.05, non-diabetic or diabetes group vs. control group. ↓ Significant decrease, *P*<0.05, diabetes group vs. non-diabetic group. ↑ Significant increase, *P*<0.05, diabetes group vs. non-diabetic group.

**Table 3:** Comparisons of microstructure, BMD and strength of subchondral bone on medial side in patients without or with T2D

Bony structure	Control (*n*=20)	Non-diabetic (*n*=70)	Diabetes (*n*=51)	*P*
(BV/TV)/%	29.18±2.51	39.47±5.05*	↓ 31.37±3.87*	**0.001**
Tb.N/mm^−1^	2.69±0.38	2.39±0.27*	↓ 2.05±0.21*	**0.002**
SMI	1.67±0.26	0.34±0.1*	↑ 0.72±0.11*	**< 0.001**
Tb.Sp/μm	204.32±35.61	326.31±76.71*	↑ 370.22±81.36*	**< 0.001**
Tb.Th/μm	108.42±13.57	174.27±25.03*	↓ 145.01±17.68*	**0.014**
Conn.D/mm^−3^	338.43±54.31	120.29±21.32*	↓81.3±17.63*	**0.012**
BMD/(mg·cm^−^^3^)	682.22±43.77	802.51±96.41*	↓ 664.67±87.23	**0.023**
Pl.Th/mm	0.84±0.16	1.18±0.36*	↓ 0.86±0.15	**0.036**
Pl.Po/%	37.78±3.53	42.77±5.56*	↑53.91±6.33*	**0.017**
Elastic modulus/MPa	497±52	1041±175*	↓659±65	**0.033**

Abbreviations: ANOVA, analysis of variance; BMD, bone mineral density; BV/TV, bone volume fraction; PI.Po, subchondral plate porosity; PI.Th, subchondral plate thickness; SMI, structure model index; T2D, type 2 diabetes; Tb.N, trabecular number; Tb.Th, trabecular thickness; Tb.Sp, trabecular separation.

The comparisons of microstructure parameters among the three groups were performed using one-way ANOVA and expressed as mean±s.d. A *post-hoc* test was further performed if the result was significant. Bold text indicates a statistically significant difference with a *P-*value<0.05. *P<0.05, non-diabetic or diabetes group vs. control group. ↓ Significant decrease, *P*<0.05, diabetes group vs. non-diabetic group. ↑ Significant increase, *P*<0.05, diabetes group vs. non-diabetic group.
