# Supplementary material for: Abnormal subchondral bone remodeling and its association with articular cartilage degradation in knees of type 2 diabetes patients
Source: Bone Res. 2017 Nov 7;5:17034–. doi: 10.1038/boneres.2017.34 (PMC5674679; doi:10.1038/boneres.2017.34)
Supplement: Supplementary Information [file boneres201734-s1.docx]

| **Diabetic medications** | **Non-diabetic**  **(n = 70)** | **Diabetes**  **（n = 51）** |
| --- | --- | --- |
| Biguanides (e.g., metformin) – n (%) | 0 (0.0) | 43 (84.3) |
| Insulin – n (%) | 0 (0.0) | 20 (39.2) |
| Sulfonylureas – n (%) | 0 (0.0) | 8 (15.8) |
| Dipeptidyl peptidase-4 (DPP-4) inhibitors – n (%) | 0 (0.0) | 0 (0.0) |
| α-glucosidase inhibitors – n (%) | 0 (0.0) | 0 (0.0) |
| Glucagon-like peptide analogs and agonists – n (%) | 0 (0.0) | 0 (0.0) |
| Meglitinides – n (%) | 0 (0.0) | 0 (0.0) |
| Sodium glucose co-transporter-2 (SGLT-2) inhibitors – n (%) | 0 (0.0) | 0 (0.0) |

**Supplementary table 1** Diabetic medications of the patients with type 2 diabetes

Note that patients who were on thiazolidinediones had been excluded from this study as it has been reported that the use of thiazolidinediones contributes to bone loss in T2D (see *patients and methods* and *discussion* section).
